# Videodermoscopy Does Not Enhance Diagnosis of Scalp Contact Dermatitis Due to Topical Minoxidil

**DOI:** 10.4103/0974-7753.58557

**Published:** 2009

**Authors:** Antonella Tosti, Aline Donati, Colombina Vincenzi, Gabriella Fabbrocini

**Affiliations:** Department of Dermatology, University of Bologna, Italy,; 1 Department of Dermatology, University of San Paulo, Brazil,; 2 Section of Dermatology, Department of Systematic Pathology, University of Naples Federico II, Naple, Italy

**Keywords:** Dermoscopic patterns, minoxidil, scalp allergic contact dermatitis, videodermoscopy

## Abstract

**Background:**

Videodermoscopy (VD) is a noninvasive diagnostic tool that provides useful information for the differential diagnosis of scalp disorders.

**Objectives:**

The aim of this study was to investigate if dermoscopy may help the clinician in the diagnosis of contact dermatitis of the scalp.

**Materials and Methods:**

We analyzed the dermoscopic images taken from 7 patients with contact dermatitis due to topical minoxidil, 6 patients complaining of intense scalp itching during treatment with topical minoxidil but with negative patch tests and 19 controls. The following dermoscopic patterns described for scalp diseases were evaluated: Vascular patterns (simple loops, twisted loops and arborizing lines), follicular/perifollicular patterns (yellow dots, empty ostia, white dots, peripilar signs), white scales, yellow scales, follicular plugging, hair diameter diversity, honeycomb pattern and short regrowing hairs. Findings were graded from 0-4, according to severity in 20-fold magnifications. Statistical analysis included univariate analysis and Chi-square test by SPSS version 12.

**Results:**

There were no statistical differences in the analysis of the vascular patterns and scales between the 3 groups.

**Conclusions:**

We were not able to detect dermoscopic features that can help the clinician in distinguishing scalp contact dermatitis due to topical minoxidil from other conditions that cause severe scalp itching. In particular, minoxidil contact dermatitis does not produce increase or alterations in the morphology of the scalp vessels or significant scalp scaling when evaluated with dermoscopy.

## INTRODUCTION

The scalp is frequently exposed to chemical compounds which contain potential contact allergens.[1] Most cases of allergic contact dermatitis of the scalp (ACDS) are due to cosmetics, particularly hair dyes; but topical drugs, including topical minoxidil, can also be responsible.[2]

Diagnosis of minoxidil contact dermatitis always requires patch-testing as clinical signs are usually mild and most patients only complain of persistent itching, which is often wrongly attributed just to scalp irritation.[3] Patients with scalp itching during topical minoxidil treatment should undergo patch-testing with the minoxidil lotion (2% or 5% in propylene glycol, ethanol and water) and propylene glycol 5% in petrolatum and 30% in water as testing minoxidil in other vehicles may yield false-negative results.[4]

Videodermoscopy (VD) is a noninvasive diagnostic tool already shown to be very useful in the differential diagnosis of scalp disorders.[5,6] Although primarily used to investigate alopecia or hair shaft disorders, VD vascular pattern analysis has recently shown promising results in inflammatory diseases such as psoriasis, both in the scalp and in other body areas.[7‐10]

The aim of this study was to investigate if dermoscopy may help the clinician in the diagnosis of contact dermatitis due to topical minoxidil.

## MATERIALS AND METHODS

This study was performed on the dermoscopic images taken from 13 female patients complaining of scalp itching of recent onset during treatment with 2% topical minoxidil. None was affected by seborrheic dermatitis and all complained that itching was persistent and severe and did not improve after shampooing. Clinical inspection of the scalp did not show signs of acute or chronic inflammation such as erythema, vesicles or scaling.

All patients were followed by our consultation for hair disorders and had been using a commercial preparation containing 2% topical minoxidil twice a day for androgenetic alopecia (AGA) or biopsy-proven alopecia areata incognita (AAI). All patients were patch-tested with the Italian standard SIDAPA series, 2% minoxidil solution in propylene glycol and alcohol and with propylene glycol 5% in petrolatum. Seven patients had a positive ++ reaction to the minoxidil solution at 72 hours. Data on diagnosis, duration of minoxidil treatment and results of patch tests are reported in Table 1.

**Table 1:** Clinical data and patch test results of the 13 female patients with scalp itching

	AGA	AAI	Duration of treatment (mean)	Duration of itching (mean)
Positive patch test	4	3	5 to 26 mths (12 mths)	12 to 30 days (16 days)
Negative patch test	5	1	3 to 17 mths (7 mths)	30 to 102 days (98 days)

AGA = Androgenetic alopecia; AAI = Alopecia areata incognita

In all patients, we analyzed the dermoscopic images taken at the moment of consultation for scalp itching. Images were acquired using a computerized polarized-light videomicroscopy system (FotoFinderdermoscope, Teachscreen Software, Bad Birnbach, Germany), at magnifications ranging from 20× to 70×. A water interface solution was applied prior to the acquisition of each epiluminescent image.

The dermoscopic images taken from the scalp of 19 volunteers who did not complain of scalp itching were used as controls. These included 6 patients with androgenetic alopecia who were not utilizing topical minoxidil and 13 subjects unaffected by hair or scalp disorders.

Videodermoscopy images of the 5 areas from the frontal and parietal scalp were analyzed for dermoscopic patterns previously described for scalp diseases.[4] These included vascular patterns (simple loops, twisted loops and arborizing lines), follicular/perifollicular patterns (yellow dots, empty ostia, white dots, peripilar signs), white scales, yellow scales, follicular plugging, honeycomb pattern and short regrowing hairs and hair diameter diversity. Findings were graded from 0-4, according to severity in 20-fold magnifications.

Statistical analysis included univariate analysis and Chi-square test by SPSS version 12.

After univariate analysis, which showed the distribution of dermoscopy data, we graded every observed dermoscopy parameter as follows: If equal or < 2 absent and if > 2 present; and we cross-tabulated with the diagnosis, as listed in [Tables 2–4]. Most AGA patients of the control group had initial AGA and their hair diameter diversity was scored 'less than 2.'

**Table 2:** Presence of each dermoscopy parameter for diagnosis in patients affected by allergic contact dermatitis

	S_2_	T_2_	AI_2_	Ostia_2_	Peripil_2_	Ydots_2_	Wd_2_	Ws_2_	Ys_2_	H_2_	D_2_	Ho_2_	Sh_2_
AAI	0	0	0	0	0	100%	0	0	0	0	0	0	100%
AGA	0	0	0	0	0	0	0	0	0	0	100%	0	0

AGA = Androgenetic alopecia; AAI = Alopecia areata incognita

**Table 3:** Presence of each dermoscopy parameter for diagnosis in patients affected by scalp itching

	S_2_	T_2_	AI_2_	Ostia_2_	Peripil_2_	Ydots_2_	Wd_2_	Ws_2_	Ys_2_	H_2_	D_2_	Ho_2_	Sh_2_
AAI	0	0	0	0	0	0	0	0	0	0	0	0	0
AGA	0	0	25%	100%	0	0	0	0	0	0	100%	0	60%

AGA = Androgenetic alopecia; AAI = Alopecia areata incognita

**Table 4:** Presence of each dermoscopy parameter for diagnosis in controls

	S_2_	T_2_	AI_2_	Ostia_2_	Peripil_2_	Ydots_2_	Wd_2_	Ws_2_	Ys_2_	H_2_	D_2_	Ho_2_	Sh_2_
AGA	0	0	0	0	0	0	0	0	0	0	17%	33%	0
Healthy	0	0	0	0	0	0	0	0	0	0	0	0	0

S_2_ = Simple loops present; T_2_ = Twisted loops present; AI_2_ = Arborizing lines present; O_2_ = Empty ostia present; Peripil_2_ = Peripilar signs present; Ydots_2_ = Yellow dots present; Wd_2_ = White dots present; Ws_2_ = White scales present; Ys_2_ = Yellow scales present; H_2_ = Follicular plugging present; D_2_ = Hair diameter diversity present; Ho_2_ = Honeycomb pattern present; Sh_2_ = Short regrowing present, AGA = Androgenetic alopecia; AAI = Alopecia areata incognita

## RESULTS

There were no statistical differences in the analysis of the vascular patterns between the 3 groups.

The only finding that was significantly associated with scalp allergic contact dermatitis was an increased number of yellow dots (*P* < 0.005).

This association was present only in patients with AAI and allergic contact dermatitis (ACD) and was then considered a discriminating parameter for ACD. Short regrowing hairs were variously present in AAI and AGA with and without ACD and for this reason were not considered as a discriminating parameter for ACD.

Patients with scalp itching and negative patch tests showed an increased number of empty ostia (*P* < 0.05) and short regrowing hairs (*P* < 0.05).

The honeycomb pattern was more frequently seen in the control group than in the study group (*P* < 0.05).

## DISCUSSION

Videodermoscopy (VD) is a noninvasive tool recently used for diagnosis and follow-up of many scalp and hair shaft disorders.[5] Recently, VD has also been proven to be helpful in distinguishing psoriasis from lichen planus[10] and for diagnosis of nail bed psoriasis,[8] palmar and plantar psoriasis[7] and plaque-type psoriasis.[9] In all of these studies, vascular patterns were the most significant abnormalities. VD alterations of seborrheic dermatitis, which is the most common eczematous disorder in the scalp, include diffusely distributed interfollicular twisted red loops and white epidermal scale.[5,11]

There are no studies published on dermoscopy of contact dermatitis in PubMed database.

According to the pathophysiology of contact dermatitis and the literature on VD in other types of inflammatory disorders, we expected to find differences in the vascular and scale patterns between allergic contact dermatitis and other scalp conditions associated with itching or control scalp. After statistical analysis, however, we did not find significant differences in the vascular patterns or in the degree of scaling.

On the other hand, we found a statistically significant increase in the number of empty ostia and shorter growing hairs in patients with scalp itching when compared with controls and the contact dermatitis group. As the duration of scalp itching was significantly longer in these patients as compared to those in the contact dermatitis group, these findings may just confirm the hypothesis that chronic scalp inflammation may increase hair loss. We did not find these features in patients with minoxidil contact dermatitis possibly because we analyzed the scalp in the acute phase and it has been shown that hair loss due to allergic contact dermatitis occurs 2 to 3 months after the acute phase.[12]

The increased number of yellow dots in patients with allergic contact dermatitis may be related to the fact that in this group we had 3 patients who were using topical minoxidil to treat alopecia areata incognita, wherein yellow dots are a typical finding; in the group of patients with scalp itching, we had only 1 patient with alopecia areata incognita.[6] The presence of the honeycomb pattern in the control group probably just indicates that controls expose their scalp to the sun more than patients with scalp disease, who are probably better oriented to protect their scalp with hats or other devices.

## CONCLUSIONS

We were not able to detect dermoscopic features that can help the clinician in distinguishing scalp contact dermatitis due to topical minoxidil from other conditions that cause severe scalp itching. In particular, minoxidil contact dermatitis does not produce increase or alterations in the morphology of the scalp vessels or significant scalp scaling when evaluated with dermoscopy.

## References

[1] Hillen U, Grabbe S, Uter W (2007). Patch test results in patients with scalp dermatitis: Analysis of data of the Information Network of Departments of Dermatology. Contact Dermatitis.

[2] Friedman ES, Friedman PM, Cohen DE, Washenik K (2002). Allergic contact dermatitis to topical minoxidil solution: Etiology and treatment. J Am Acad Dermatol.

[3] Tosti A, Piraccini BM, Tosti A, Piraccini BM (2006). Scalp contact dermatitis. Diagnosis and treatment of hair disorders: An evidence based atlas.

[4] Whitmore SE (1992). The importance of proper vehicle selection in the detection of minoxidil sensitivity. Arch Dermatol.

[5] Ross EK, Vincenzi C, Tosti A (2006). Videodermoscopy in the evaluation of hair and scalp disorders. J Am Acad Dermatol.

[6] Tosti A, Whiting D, Iorizzo M, Pazzaglia M, Misciali C, Vincenzi C (2008). The role of scalp dermoscopy in the diagnosis of alopecia areata incognita. J Am Acad Dermatol.

[7] Micali G, Nardone B, Scuderi A, Lacarrubba F (2008). Videodermatoscopy enhances the diagnostic capability of palmar and/or plantar psoriasis. Am J Clin Dermatol.

[8] Iorizzo M, Dahdah M, Vincenzi C, Tosti A (2008). Videodermoscopy of the hyponychium in nail bed psoriasis. J Am Acad Dermatol.

[9] Vazquez-Lopez F, Zaballos P, Fueyo-Casado A, Sanchez-Martin J (2007). A dermoscopy subpattern of plaque-type psoriasis: Red globular rings. Arch Dermatol.

[10] Zedlaudek I, Argenziano G (2006). Dermoscopy subpatterns of inflammatory skin disorders. Arch Dermatol.

[11] Rosina P, Zamperetti MR, Giovannini A, Girolomoni G (2007). Videocapillaroscopy in the differential diagnosis between psoriasis and seborrheic dermatitis of the scalp. Dermatology.

[12] Tosti A, Piraccini BM, van Neste DJ (2001). Telogen effluvium after allergic contact dermatitis of the scalp. Arch Dermatol.

